# The PRECISE-DYAD Neurodevelopmental substudy protocol: neurodevelopmental risk in children of mothers with pregnancy complications

**DOI:** 10.12688/wellcomeopenres.19689.2

**Published:** 2024-08-05

**Authors:** Dorcas N. Magai, Jaya Chandna, Marie-Laure Volvert, Rachel Craik, Hawanatu Jah, Fatoumata Kongira, Kalilu Bojang, Angela Koech, Grace Mwashigadi, Agnes M. Mutua, Hannah Blencowe, Umberto D'Alessandro, Anna Roca, Marleen Temmerman, Peter von Dadelszen, Amina Abubakar, Melissa Gladstone, The PRECISE DYAD Network

**Affiliations:** 1 Department of Women's and Children's Health, Institute of Life Course and Medical Sciences, University of Liverpool, Liverpool, England, L12 2AP, UK; 2 MARCH Centre, London School of Hygiene and Tropical Medicine, London, Keppel Street, WC1E 7HT, UK; 3 Department of Women's and Children’s Health, School of Life Course and Population Sciences, Faculty of Life Sciences and Medicine, King's College London, London, England, UK; 4 Nuffield Department of Women’s and Reproductive Health, University of Oxford, Oxford, England, UK; 5 Medical Research Council Unit The Gambia at the London School of Hygiene and Tropical Medicine, Fajara, The Gambia; 6 Centre of Excellence Women and Child Health, The Aga Khan University, Nairobi, Kenya; 7 Institute for Human Development, The Aga Khan University, Nairobi, Kenya

**Keywords:** Maternal health, child health, neurodevelopment, early child development, global health, pregnancy complications

## Abstract

**Background:**

Over 250 million children are not reaching their developmental potential globally. The impact of prenatal factors and their interplay with postnatal environmental factors on child neurodevelopment, is still unclear—particularly in low- and middle-income settings. This study aims to understand the impact of pregnancy complications as well as environmental, psychosocial, and biological predictors on neurodevelopmental trajectories.

**Methods:**

This is an observational cohort study of female and male children (≈3,950) born to women (≈4,200) with and without pregnancy complications (pregnancy-induced hypertension, foetal growth restriction, and premature birth) previously recruited into PREgnancy Care Integrating Translational Science, Everywhere study with detailed biological data collected in intrapartum and post-partum periods. Children will be assessed at six weeks to 6 months, 11-13 months, 23-25 months and 35-37 months in rural and semi-urban Gambia (Farafenni, Illiasa, and Ngayen Sanjal) and Kenya (Mariakani and Rabai). We will assess children's neurodevelopment using Prechtls General Movement Assessment, the Malawi Development Assessment Tool (primary outcome), Observation of Maternal-Child Interaction, the Neurodevelopmental Disorder Screening Tool, and the Epilepsy Screening tool. Children screening positive will be assessed with Cardiff cards (vision), Modified Checklist for Autism in Toddlers Revised, and Pediatric Quality of Life Inventory Family Impact. We will use multivariate logistic regression analysis to investigate the impact of pregnancy complications on neurodevelopment and conduct structural equation modelling using latent class growth to study trajectories and relationships between biological, environmental, and psychosocial factors on child development.

**Conclusions:**

We aim to provide information regarding the neurodevelopment of infants and children born to women with and without pregnancy complications at multiple time points during the first three years of life in two low-resource African communities. A detailed evaluation of developmental trajectories and their predictors will provide information on the most strategic points of intervention to prevent and reduce the incidence of neurodevelopmental impairments.

## Background

Over the past decades, there has been significant pressure to improve maternal and neonatal health, leading to improvements in both neonatal and maternal mortality ^1– 3^. Despite these gains, many children, especially in low- and middle-income countries (LMICs), are now surviving but not thriving ^4, 5^. Several studies have focussed on the links between stunting, poverty, and poor child developmental outcomes ^6– 8^; however, many other factors (particularly those linked to maternal health, pregnancy, and birth) may also play a significant role in the developmental outcomes of children living in LMICs.

Pregnancy complications such as pregnancy-induced hypertension (PIH), foetal growth restriction (FGR), and/or preterm birth (PTB) are potentially life-threatening and may have long-term impacts on the mother and child ^9– 12^. These pregnancy complications (PIH, FGR, and PTB) are interrelated ^13– 15^. While outcome studies of the long-term effects of pregnancy complications in children in LMICs are sparse, evidence from high-income countries (HICs) shows that these complications are associated with adverse neurodevelopmental and behavioural outcomes ^16– 22^.

Brand *et al.*, 2020 followed a Swedish cohort of children whose mothers had PIH and found that the offspring of these mothers had significantly lower cognitive scores, as well as a higher incidence of autism spectrum disorders and intellectual disability ^23^. Behavioural problems are also reported in children whose mothers had PIH at a 2-year follow-up ^22^. A systematic review of 38 studies in FGR reported that children with FGR born <35 weeks of gestation had cognitive impairment, behavioural problems, vision and hearing impairment compared with children without FGR ^24^. Several studies in HICs have shown the poor neurodevelopmental outcome in PTB ^25– 28^.

Although pregnancy complications are linked to adverse neurodevelopmental and neurological outcomes, which may include cerebral palsy, global developmental delay, and epilepsy, however, children survive without significant impairment. The difference in outcome may be due to the interaction between biological and environmental influences during prenatal and postnatal periods. Researchers have highlighted how biological ^29– 32^, environmental ^33^, and psychosocial factors ^32, 34– 40^ impact child development. Additionally, studies have shown sex differences among offspring of women with pregnancy complications. For instance, Limperopoulos *et al.*, 2008 and López-Hernández *et al.*, 2021 found that male preterm babies have poorer neurocognitive outcomes than their female counterparts ^25, 29^. Therefore, a child's birth outcomes ( *e.g.*, preterm birth) in the presence of other key risk factors ( *e.g.*, maternal health, birth complications, insufficient stimulation) is likely to affect developmental outcomes across the lifespan ^41^.

However, there is much less information regarding factors in pregnancy that may influence child development—particularly in low-resource settings. The sparsity of long-term neurodevelopmental follow-up of babies from pregnancy complications is due to the difficulty in identifying phenotyped cohorts of women early in the gestational period and the lack of clear, detailed information (including biological data on the mother's health) throughout these pregnancies in settings where antenatal care and documentation of that care, may be more limited. Furthermore, long-term neurodevelopmental follow-up of children is challenging due to the expense of bringing families regularly back to clinics for detailed standardised testing.

This lack of data makes it hard to estimate the extent to which pregnancy complications affect child neurodevelopment. It is imperative to bridge this data gap in settings such as sub-Saharan Africa, where there may be higher rates of pregnancy complications and environmental risks than HICs leading to poorer prognosis of children with neurodevelopmental difficulties and impairments.

### PRECISE-DYAD neurodevelopmental substudy

This neurodevelopmental substudy sits within the more extensive PREgnancy Care Integrating Translational Science, Everywhere (PRECISE)-DYAD observational cohort study, which seeks to provide an epidemiological and mechanistic understanding of maternal and infant health and development in relation to in-utero disease pathways in sub-Saharan African settings ^42^. The PRECISE-DYAD Network members are listed in Table 1.

**Table 1.** The PRECISE-DYAD network.

In-country teams	Members
THE GAMBIA: Medical Research Council Unit The Gambia at the London School of Hygiene and Tropical Medicine, Fajara	Umberto D’Alessandro, Anna Roca, Hawanatu Jah, Andrew Prentice, Melisa Martinez-Alvarez, Brahima Diallo, Abdul Sesey, Sambou Suso, Fatima Touray, Modou F.S. Ndure, Gibril Gabbidon, Yahaya Idris, Baboucarr Njie, Fatoumata Kongira, Lawrence Gibba, Abdoulie Bah and Yorro Bah.
KENYA: Aga Khan University, Nairobi	Marleen Temmerman, Angela Koech, Patricia Okiro, Geoffrey Omuse, Grace Mwashigadi, Joseph Mutunga, Isaac Mwaniki, Moses Mukhanya, Onesmus Wanje, Marvin Ochieng, Amina Abubakar, Agnes Mutua
MOZAMBIQUE : Centro de Investigação em Saúde de Manhiça, Manhiça	Esperança Sevene, Corssino Tchavana, Salesio Macuacua, Anifa Vala, Helena Boene, Lazaro Quimice, Sonia Maculuve, Ana Ilda Biza, Jovito Nunes and Charfudin Sacoor
Central co-ordinating team	
Department of Women and Children's Health, School of Life Course Sciences, Faculty of Life Sciences and Medicine, King's College London	Peter von Dadelszen, Laura A. Magee, Rachel Craik, Hiten D. Mistry, Marie-Laure Volvert, Anne Rerimoi, Giulia Ghillia, Thomas Mendy
Co-Investigator team	
Midlands State University, Zimbabwe	Prestige Tatenda Makanga, Liberty Makacha, Reason Mlambo
Kings College London	Lucilla Poston, Rachel Tribe, Sophie Moore, Tatiana Taylor Salisbury
London School of Hygiene and Tropical Medicine	Hannah Blencowe, Veronique Filippi, Joy Lawn, Jaya Chandna, Joseph Waiswa
St George's, University of London	Asma Khalil
University of British Columbia	Marianne Vidler, Jing (Larry) Li, Jeff Bone, Mai-Lei (Maggie) Woo Kinshella, Domena Tu, Akshdeep Sandhu, Kelly Pickerill
Imperial College London	Ben Barratt
University of Liverpool/Liverpool School of Tropical Medicine	Melissa Gladstone, Dorcas Magai
C-Squared Development	Chris Clarke

### Aims and objectives

This substudy aims to understand the pathway and extent of any impact of *in-utero* disease on infant neurodevelopment in The Gambia and Kenya.

Specifically, we aim first to quantify the impact of pregnancy complications on the likelihood of having a moderate to severe neurodevelopmental disability, a visual impairment, and/or epilepsy at the age of 2 to 3 years. Second, we aim to understand the relationship between pregnancy complications and neurodevelopmental outcomes at each time point, but also longitudinally, across multiple developmental domains including fine and gross motor, language, and social-emotional development. Our third objective is to understand the biological mechanisms that underpin the relationship between intrauterine and early postnatal risk factors and neurodevelopmental outcomes. The fourth objective is to explore whether environmental exposures modify neurodevelopmental outcomes ( *e.g.*, air and water) by assessing co-exposures such as nutrition, quality of care, social-economic status, maternal education, maternal depression, family care indicators, adversity measures in the home during pregnancy and perinatal period.

Understanding pregnancy complications and the associated outcomes and mechanisms through which these associations occur is critical for research, clinical practice, and policy, especially in sub-Saharan Africa, to inform the optimisation of maternal and child health care and enable children to survive and thrive.

### Theoretical framework

**Figure 1.**
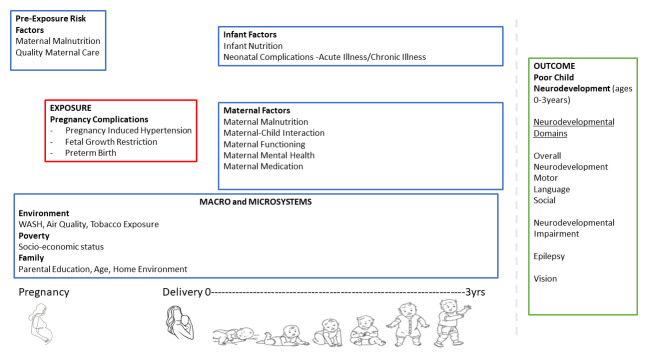
Neurodevelopmental outcomes in children of mothers with pregnancy complications – a conceptual framework. Note: WASH- Water Sanitation and Hygiene.

According to the recently released Lancet series on optimising children and adolescents' health, a child's birth outcomes ( *e.g.*, preterm birth) in the presence of other key risk factors ( *e.g.*, maternal health, birth complications, insufficient stimulation) will likely affect developmental outcomes across the lifespan ^41^. We developed our framework based on this concept - where the interaction of biological, environmental, and psychosocial factors will affect child development ( Figure 1). Pregnancy complications (PIH, FGR, PTB) are associated with biological factors (maternal physical health, perinatal complications, child's malnutrition, sex). These may also further interact with environmental factors (air quality, water sanitation and hygiene, and exposure to tobacco) as well as psychosocial factors (quality of maternal care, maternal mental health, maternal functioning, child stimulation, socioeconomic status, and home environment), which may then influence child developmental outcomes (fine motor, gross motor, social, emotional, and physical) and risk for impairment (neurodevelopmental and vision impairment, and epilepsy). Furthermore, pregnancy complications are associated with psychosocial factors such as depressive and anxiety symptoms ^38^, which may influence child development.

## Protocol

### Study design

This is an observational study of a cohort of women (≈4,200) and their male and female children (≈3,950) in The Gambia and Kenya. The participants were previously recruited into the PRECISE study ^43^ at any time of their pregnancy, and they will be followed up for three years. It is estimated that the prevalence of PIH, FGR and PTB in sub-Saharan Africa is 8% ^13^, 16.5% ^44^ and 12% ^45^, respectively. Compared with a control group, a large effect size (0.7) was observed in neurocognitive delay in children whose mothers had PIH ^46^. Small effect sizes were observed in cognition (0.3) in FGR ^47^. A large effect size was observed in cognition (0.7), while small effect sizes were observed in motor (0.4) in PTB ^16^. We computed the sample sizes needed in each group based on these effect sizes. Using G*power 3.1 software calculations ^48^, at least 50 participants in the PIH group, 250 in FGR and 150 participants in the PTB are required to give a power of 95% (alpha = .05) to detect significant differences between these groups and a control. Therefore, a sample size of at least 300 participants for each group (a total of at least 900 cases and 900 age-matched controls) will be considered adequate based on the recommended sample size for SEM considering a 10% loss to follow-up of the original sample. The consent process, confidentiality, community engagement, and ethical approval details are described elsewhere ^42^.

### Research setting

As described below, we will conduct this study across our two linked sites.

The Gambia: The study is at the MRC Unit The Gambia at the London School of Hygiene and Tropical Medicine (MRCG at LSHTM). Field research will occur at the Maternal Newborn Child and Adolescent Health Clinic in Farafenni (urban primary health centre (PHC)), associated rural PHCs in Illiasa and Ngayen Sanjal and the Farafenni General Hospital.

Kenya: The study is based at Aga Khan University, Nairobi. The field research will be conducted in peri-urban Mariakani Subcounty Hospital and rural Rabai Subcounty Hospital.

### Data collection procedures

**Figure 2.**
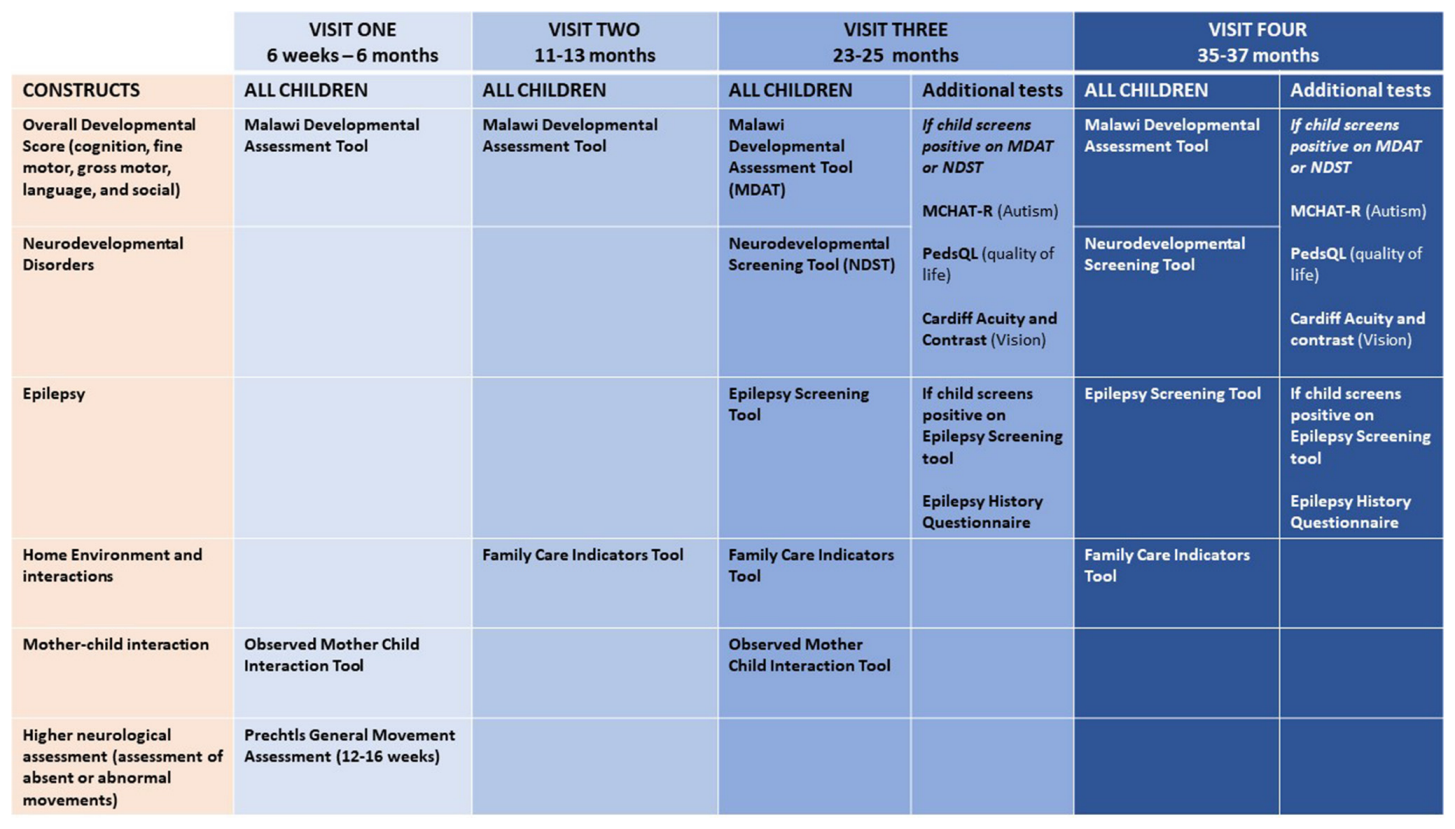
Data collection process. Note: MDAT- Malawi Developmental Assessment Tool, NDST- Neurodevelopmental Screening Tool, MCHAT-R -Modified Checklist for Autism in Toddlers Revised, PedsQL Pediatric Quality of Life Inventory Family Impact Module.

Data will be collected from mothers and infants across four-time points to provide information on children's neurodevelopmental trajectories visit 1 (6 weeks to 6 months) visit 2 (11–13 months), visit 3 (23–25 months), and visit 4 (35–37 months). These assessments are on-going and are administered at each visit as summarised in Figure 2. We outline the different assessments conducted across these visits below:

***Malawi Developmental Assessment Tool (MDAT) (Visit 1, 2, 3, and 4).*** The primary outcome measure is the MDAT ^49^, which has been used in several LMICs. The MDAT has 157 items (these are age-related items, so only a subset of these items is asked, depending on the child's age) that measure four different domains; gross motor, fine motor, language, and social. The MDAT gives a total developmental score and individual domain scores for the four domains. Three of the four domains are assessed by direct observation of the child, and the fourth domain (social) is a questionnaire administered to the caregiver. Assessors fluent in the local language and familiar with the cultural context underwent virtual and in-person training and standardisation procedure. The tool has been used across cultures in LMICS and has good psychometric properties (reliability with kappa >0.4 in most items; and excellent diagnostic accuracy – specificity = 82% and sensitivity = 97%) ^49^.

Since the MDAT was originally developed in Malawi (with almost a similar context to Kenya and The Gambia), almost all the items were retained for both sites. We made a few changes in the Gambia; for example, mothers were asked if children used sand instead of clay to make toys. This is because clay is not common as the area is situated in the Sahel zone, and sand is most common.

***Prechtls General Movement Assessment (Visit 1).*** Infants whose general movements are absent or abnormal are at higher risk of neurological conditions, particularly cerebral palsy. The General Movements Assessment ^50^ is used to identify absent or abnormal general movements, and depending on the type of general movements, the abnormality can be highly predictive of cerebral palsy by about 3 months of age ^50^.

The assessment will be done on children between 12 to 16 weeks after birth and consists of recording a short video of the baby's spontaneous movements. General movements are assessed with the infant awake and lying on their back while they are calm and alert. The infant should not have toys or pacifiers; parents could be watching nearby but not interacting with their baby. The baby is videoed for 3–5 minutes, and the assessment is scored from the video. The tool has excellent diagnostic accuracy (sensitivity = 97%; specificity = 89%) ^51^.

***Observervation of Mother-Child Interaction (OMCI) (Visit 1, 3, and 4).*** Maternal-child interaction is measured by the OMCI tool ^52^. The OMCI tool is a simple rating scale that is measured at the time of assessment by a trained local culturally embedded observer. It measures the following: maternal sensitivity, acceptance and responding, scaffolding, language stimulation and mutual enjoyment, which are assessed during a short interaction. The tool includes scoring based on observations such as the mother touching the child or helping the child build on their learning, smiling, and interacting with the mother. In visit 1 the caregiver is offered a few toys (rattle, ball, book, and doll) and is instructed to play with their child for 3 minutes as they would at home. In visit 3 the caregiver is offered only a book and is instructed to play with the child for 5 minutes. The scoring is based on a 5-point Likert scale (never, very few, sometimes, 5 or more times). This tool has high inter-observer reliability ( *r* = 0.85) ^53^ and has been used in other LMICS settings ^52^.

Given the size of the study and the number of sites, we were keen to video OMCI assessments to ensure the reliability of scoring. However, the assessment team noted that video recording did not provide a natural environment for the mother and the child to interact as they would at home. The cameras made the mothers uncomfortable, affecting how they interacted with their children. Therefore, the video recording was stopped to provide a comfortable environment for maternal and child interaction, which instead was done by direct observation with well-trained assessors. To enhance the accuracy of the mother-child ratings, every 10 ^th^ dyad assessment was jointly evaluated by a senior research officer and a research officer. They compared their scores and discussed any discrepancies to ensure high inter-rater reliability.

Additionally, the assessors noted that at visit 3 (23–25 months), the presence of more than one stimulus (book, ball, doll, rattle, and toy car) for play confused the mother and the child. It was decided that only one stimulus (a book) would be used as it worked better for the mothers and their children. Therefore, the dyad was presented with only one play item as an option.

***Neurodevelopmental Disorder Screening Tool (NDST) (Visit 3 and 4).*** The NDST ^54^ is a 39 question screening tool for various neurodevelopmental disorders (NDDs). NDDs are disabilities associated primarily with the neurological system and brain functioning. NDDs screened by the NDST include the following: intellectual disability (ID), communication disorders, autism spectrum disorder (ASD), attention-deficit/hyperactivity disorder (ADHD) and neurodevelopmental motor disorders. Other neurological impairments that are assessed using NDST include visual impairment, hearing impairment, and epilepsy. The tool has been used in Kenya and has excellent diagnostic accuracy (specificity = 82.8%; sensitivity = 87.8%) ^55^.

***Epilepsy Screening Tool (Visit 3 and 4).*** The epilepsy screening tool is a 10-item questionnaire administered to caregivers to report if their child shows any signs of epilepsy. The caregiver responds with a "YES" or a "NO". If a caregiver answers NO to the first four questions, the rest are not asked, as the latter six questions are asked further to understand the types of seizures in a child. If a child screens positive, they receive an additional set of questions from the Epilepsy History Questionnaire, a 70-item tool designed to assess further and classify the type of epilepsy in patients who experienced epileptic seizures in the past ^56^.

For visits 3 and 4, if a child is screened positive (meeting a requirement for further testing) on either the NDST or MDAT, they receive the following additional tests:

***Modified Checklist for Autism in Toddlers Revised (M-CHAT-R) (Visit 3 and 4).*** M-CHAT-R is a 20-item checklist used to screen for autism in toddlers aged 18–30 months ^57^. It is a parent report-based ASD screening tool to improve the early detection of ASD in the general population. The tool has excellent psychometric properties (sensitivity = 0.97; specificity = 0.95) ^58^. Children who score NO on all items except 2, 5, and 12 and those who score YES on items 2, 5, and 12 screen positive at risk of ASD.

***Pediatric Quality of Life Inventory *™* (PedsQL *™*) Family Impact Module (Visit 3 and 4).*** The (PedsQL *™*) ^59^ is a 36-item questionnaire that assesses the child's home environment and interactions that the child has with other members of the household, for example, playtime and language development. The first questions ask about the child's environment and the objects the child can learn and play with (for example, toys and books). The last questions ask about the activities members of the household carried out with the child in the past three days. This tool has excellent psychometric properties (α > 0.97) ^59^.

***Cardiff Tests (visit 3 and 4).*** We used the Cardiff acuity cards to measure the ability of a child to distinguish details of and object at a given distance and the Cardiff contrast sensitivity cards to measure the ability of the child to detect objects not clearly defined from the background. The principle of the tests is that of preferential looking ^60^. In the Cardiff Tests, each target is positioned either at the top or bottom of the card. If the target is visible, the child will look towards it, and the examiner, watching the child's eye movements, can judge the position of the target from those eye movements. These Cardiff cards have been used in LMICS such as Kenya ^61^ and Malawi ^62^.

### Assessment of risk factors

For the following risk factors, we will obtain data from PRECISE study ^63^ and other sub-studies within PRECISE-DYAD ^42^. Here, we provide a brief overview of the data collection methods. Detailed information about these sub-studies is available in the PRECISE-DYAD protocol .

Demographics: We collected information on child’s sex, maternal education, and maternal age

Socio-economic status: The caregivers' socio-economic status was assessed using the Grameen Progress out of Poverty Index ^64^, a tool that can quantify the share of program participants living below the poverty line, assess the performance of the intervention among the poor, and track poverty levels over time.

Maternal and child nutrition: The mothers provided information on the different foods they consume at home and the types of diet they follow for their children. Questionnaire-based data were collected on breastfeeding practices, including the age of cessation of exclusive breastfeeding and types of weaning foods given. Mothers were also asked whether their babies were breastfeeding. Additionally, comprehensive data on dietary intake and nutritional status was collected on both mothers and their infants. For women, this included questionnaire-based data on food security and dietary diversity and physical measures of weight, height and mid-upper arm circumference (MUAC). Biological samples have also been collected and biobanked for future analyses of biomarkers of micronutrient status, including iron, folate, iodine, and vitamins A, D and B12. For infants, this included questionnaire-based data on infant feeding practices and dietary intake and measures of weight, length, MUAC and head circumference at 6 weeks to 6 months, 12 months, 24 months and 36 months of age.

Maternal medication: Mothers were asked if they were taking any medication for blood pressure, diabetes, thyroid supplements, anti-seizure medication, antiretroviral, antibiotics, or aspirin during pregnancy. 

Quality maternal Care: The quality of care was assessed using a questionnaire in which mothers reported their experiences at the facility during delivery, after delivery, during admission, and after discharge. The mothers were also asked if they experienced any form of disrespect during delivery and about the care their children received postdelivery.

Maternal mental health: The Generalised Anxiety Disorder 7 (GAD-7) ^65^ was used to assess the mothers' feelings of nervousness, worry, lack of control, inability to relax, restlessness, and irritability. The tool assesses the participants' feelings about nervousness, worries, anxiousness, loss of control, inability to relax, restlessness, and irritability. The responses are measured on a 4-point Likert scale.

Patient Health Questionnaire-9 (PHQ-9). The PHQ-9 is a depression module based on the Diagnostic Systematic Manual criteria and assesses participants' mental health in the previous two weeks ^66^. The mothers responded to questions regarding their mental wellbeing by rating them from 0 to 3, based on how accurately the statements reflect their current situation.

Posttraumatic stress disorder checklist-civilian version (PCL-C). The PCL-C is a 17 item self-report measure that assesses the 20 diagnostic systematic manual (DSM-5) symptoms of PTSD ^67^. The mothers were asked to rate how well the statements reflected their well-being over the past month.

Maternal functioning: This was assessed using the WHO Disability Assessment Schedule (WHODAS). WHODAS was designed as a general measure to assess health, functioning, and disability. The tool measures 6 domains of function, including cognition, mobility, self-care, getting along, life activities, and participation ^68^.

Information on sanitation, hygiene (WASH), and air quality: In households where women consented to participate, water samples were collected to assess bacterial contamination, alongside a comprehensive questionnaire focusing on the home's water WASH. The questionnaire covered aspects such as drinking water, availability of toilet facilities, and hand-washing experiences. Data on air quality were collected through personal and fixed environmental measurements, aiming to quantify the primary sources of sources of ambient and residential air pollution.

Tobacco exposure: Mothers are asked if they used any tobacco products. This includes the type of tobacco use, duration, and frequency of use.

Home environment: The family indicator questionnaire was used to assess the child's home environment and interactions with other household members, such as playtime and language development. The first questions ask about the child's environment and the objects the child has access to learn and play with (for example, toys and books). The last questions ask about the activities members of the household carried out with the child in the past three days.

Neonatal complications: Obstetric history was captured using 27 questions centered on labour (e.g duration of labour) and delivery events (e.g mode of delivery). Additionally, postdelivery maternal and baby's health were documented in the PRECISE study ^63^.

### Adaptation of assessments

Although some of the tools used in this study ( *e.g.*, MDAT and NDST) were developed in LMICs, most were developed in HICs and adapted to suit the cultural differences in these two LMICs. All tools were forward and back translated to ensure accuracy. During training, the team also suggested areas on the neurodevelopmental questionnaires that needed to be revised or worded differently to bring out the meaning as intended.

### Training activities

The teams in both sites were trained to conduct the neurodevelopmental assessments in two phases. Due to COVID-19-related travel restrictions, the first phase involved online training for the neurodevelopmental assessments for visits 1 and 2. This training involved four participants (two in each site) who were trained and certified to train the other assessors. The training was conducted in eight sessions and lasted for four days. The trainers were experts in child development (child psychologist and a neurodevelopmental paediatrician). After completing the training and before certification, the trainees practised and took videos of how they did the assessments and scored the MDAT tool. The trainers scored these videos, and it was only after certification that the trainees were able to train the rest of the team. The main trainers supervised these trainings sessions. The second phase of training was in-person. Psychologist experts in child development conducted this training. The team was trained on the neurodevelopmental assessments for visits 3 and 4. The training was first done in The Gambia and then in Kenya.

### Quality control

In this study, quality control measures will be implemented throughout the data process. Three main methods to ensure quality checks during the assessments are used, including:

○ *Observation/shadowing:* The existing MDAT assessment team invites the newly trained team to observe the NeuroDev assessment for at least one month before being certified to conduct the assessments.
○ *Inter-rater assessments and Double scoring:* For each site, the supervisors complete double scoring for at least one in every 20 NeuroDev assessments to ensure an inter-rater agreement between the assessors.
○ *Monthly meetings:* These regularly monthly virtual meetings ensure that the psychologist expert supports both teams in any challenges encountered during the assessments. Clarifications about any questions arising from the neurodevelopmental assessments and field activities are discussed and clarified.
○ Maintaining standardisation of items

Throughout the assessments, the team maintained standardisation of items in both sites. Whenever a toy was replaced (when damaged), the team ensured that the item met the standards and measurements as needed across all the sites.

### Data analysis plans

We will conduct and present descriptive statistics about the cohort. We will check for multicollinearity between the independent variables by comparing the pairwise correlation coefficients. Covariates with a correlation of less than 0.5 will be used in subsequent models and analyses. For the first general objective, we will compare the trajectories of MDAT across the four assessments between the exposed and unexposed groups using growth curve modelling taking into account the effects of sex. Specifically, we will use latent class growth analysis (LCGA) using structural equation modelling. For the second objective, we will use multivariate logistic regression analysis to investigate the impact of pregnancy complications on neurodevelopmental outcomes across the four domains. At this stage, we will consider all the potential covariates (independent variables) that might influence the outcome. These will include variables that we know may influence child development, such as sex, socioeconomic status, maternal education and family care indicators (stimulation in the home).

For objectives 3 and 4 we will also use structural equation modelling to understand the relationships between biological, environmental, and psychosocial factors on child development at age 3. Specifically, path analysis will be used to examine the hypothesised relationship and estimate the significance and magnitude of the hypothesised causal relationship.

### Ethics and dissemination

Approval for the PRECISE-DYAD study was obtained from King's College London (Ref HR-20/21-19714), The Gambia Government/MRC Joint Ethics Committee (Ref 22843), Aga Khan University, Nairobi Institutional Ethics Review Committee (Ref 2021/IERC-08), The National Commission for Science, Technology, and Innovation (license no. NACOSTI/P/22/18706) and University of British Columbia (Ref H20-02769). Participants gave written informed consent to take part in the study. The detailed ethical consideration of this study is highlighted elsewhere ^42^.

### Impact of COVID-19 on assessments

Most of the research activities were slowed down during the COVID-19 pandemic. First, travel restrictions made in-person training impossible. Therefore, the trainings during the first and second visits were done online. Second, adherence to COVID standards, *e.g.*, social distancing, meant that only a few participants could be recruited and assessed, which slowed down the project.

## Discussion

This cohort of mother-child dyads with and without pregnancy complications across two African countries presents a unique opportunity to further understand neurodevelopment in the first three years of life. Studies with detailed information about antenatal and perinatal periods are sparse, particularly in mothers with pregnancy complications. The PRECISE-DYAD neurodevelopment substudy presents an opportunity to understand the impact of pregnancy complications on neurodevelopment whilst also considering the many factors that are known to put children at risk for poor outcomes, *e.g.*, nutrition, maternal and family psychosocial factors, sociodemographic and environmental influences.

This rich dataset, which uses measures appropriate for the context, is critical to understanding the underpinning of neurodevelopment, particularly in the first three years of life. This substudy offers the opportunity to collect data across multiple time points to understand developmental trajectories, allowing for further understanding of the infant's brain during this period of rapid development.

As the Sustainable Development Goals(SDG) shift focus from solely survival to surviving and thriving, this study will provide important information beyond the perinatal period to longer terms outcomes of children, inclusive of screening for neurodevelopmental disabilities for which there is very little literature.

## Study status

The PRECISE-DYAD Neurodevelopmental sub study started in July 2021, but data analysis has not yet been performed. This study will continue until July 2024. The neurodevelopmental assessments at visits 1, 2, 3, and 4 are still ongoing in Kenya. In The Gambia, the visits 1 and 2 have been completed, while follow-ups for visits 3, and 4 are ongoing. However, before the end of field activities in December 2023 in The Gambia, there will be a subset of children born to PRECISE participants who had recently become pregnant and delivered a second or third time who will be followed.

## Data Availability

No data are associated with this article.
