# Supplementary material for: The PRECISE-DYAD Neurodevelopmental substudy protocol: neurodevelopmental risk in children of mothers with pregnancy complications
Source: Wellcome Open Res. 2024 Aug 5;8:508. Originally published 2023 Nov 9. [Version 2] doi: 10.12688/wellcomeopenres.19689.2 (PMC11316179; doi:10.12688/wellcomeopenres.19689.2)
Supplement: Peer review history

# Reviewer report: Reviewer response for version 2

**DOI:** 10.21956/wellcomeopenres.25204.r92166

**Published:** 2024-08-09

**Authors:** Matthew Bluett-Duncan

**Affiliations:** 1 The University of Manchester, Manchester, England, UK

I am grateful for the authors' considered responses to my feedback and have no further concerns regarding this manuscript.

Is the study design appropriate for the research question?

Yes

Is the rationale for, and objectives of, the study clearly described?

Yes

Are sufficient details of the methods provided to allow replication by others?

Yes

Are the datasets clearly presented in a useable and accessible format?

Not applicable

Reviewer Expertise:

Developmental Psychology, Teratology, Pregnancy Pharmacovigilance.

I confirm that I have read this submission and believe that I have an appropriate level of expertise to confirm that it is of an acceptable scientific standard.

# Reviewer report: Reviewer response for version 2

**DOI:** 10.21956/wellcomeopenres.25204.r92167

**Published:** 2024-08-09

**Authors:** Kimford Meador

**Affiliations:** 1 Stanford University School of Medicine, Palo Alto, CA,, USA

The revision addresses all my concerns. I fully approve the manuscript.

 Kimford J Meador, MD

Is the study design appropriate for the research question?

Yes

Is the rationale for, and objectives of, the study clearly described?

Yes

Are sufficient details of the methods provided to allow replication by others?

Partly

Are the datasets clearly presented in a useable and accessible format?

Not applicable

Reviewer Expertise:

Behavioral neurology, epilepsy, and neurodevelopmental effects of antiseizure medications and other factors

I confirm that I have read this submission and believe that I have an appropriate level of expertise to confirm that it is of an acceptable scientific standard.

# Reviewer report: Reviewer response for version 1

**DOI:** 10.21956/wellcomeopenres.21809.r80149

**Published:** 2024-05-13

**Authors:** Matthew Bluett-Duncan

**Affiliations:** 1 The University of Manchester, Manchester, England, UK

This study addresses an important gap in our understanding of the antenatal factors that may influence  infant neurodevelopment in low- and middle-income countries (LMICs). The authors correctly highlight that while there have been huge improvements in reducing neonatal and maternal morbidity in LMIC settings, there is still a long way to go to understand and improve the long-term neurodevelopmental outcomes that have a significant impact on an individual's quality of life. This study aims to begin to address this gap by recruiting and following-up a large sample of pregnant women and their children in two resource constrained settings. Importantly, the study will consider the interaction between antenatal and postnatal factors and their association with a variety of neurodevelopmental outcomes across the first 3 years of life. The results from this study are likely to provide key insights into those children who are at higher risk of neurodevelopmental impairments and to identify key opportunities for early intervention to ameliorate these effects. 

 The authors set out a clear framework and evidence base from which their protocol has been developed. Consideration has been given to the sample size and the study appears to be adequately powered to answer the research questions posed. The protocol itself provides a good level of detail regarding the study processes, including an overview of the assessment tools utilised and how they have been/will be adapted for use in these specific contexts. It is clear that considerable consideration has been given to which tools are appropriate for use in these settings and what adaptations are required to ensure that data collected are reliable and valid. 

 I only have some minor suggestions/concerns:

- The phrasing of the second sentence of the abstract "the impact of prenatal factors **then influenced by postnatal environmental factors..." **is not very clear. Maybe something along the lines of "prenatal factors and their interaction with postnatal environmental factors..." would be better.
- In the third paragraph of the background section the sentence "lower cognitive scores, autism, and intellectual disability" reads as if autism and ID were lower in children of mothers with PIH, which I assume isn't what is intended?
- In aims and objectives, the inclusion of Epilepsy as a condition to study seems to come out of the blue. While I agree it is an important disorder to include, it may be helpful to include some justification as to why it is included specifically.
- Although it is clarified later on, it was not immediately clear to me how Aim 2 is distinct from Aim 1. It may be helpful to clarify further at this point.
- For the observed mother-chid interaction the authors state that due to concerns regarding the validity of the data when using cameras to film the assessments they instead chose to rate the interactions in the moment. In my experience, rating these interactions takes quite a bit of time and requires re-watching the interactions multiple times as part of the process and so I am a little concerned about the quality of these ratings done during the assessment. It may be that this specific rating scale is more simple than the scales I have experience with and my concern is unfounded. However, I think it would be helpful to further clarify how the team have ensured that these ratings are valid and reliable beyond the statement that the assessors were "well-trained".
- I was also a bit unclear as to whether the follow-up element of the study was already ongoing or not. I recognise that this is clarified at the very end of the protocol but it may be helpful to have a comment earlier on in the document stating this very briefly.

Overall, I believe this is a well-designed study addressing an important and under-researched area that will be of huge benefit pregnant women, their children, and their healthcare professionals.

Is the study design appropriate for the research question?

Yes

Is the rationale for, and objectives of, the study clearly described?

Yes

Are sufficient details of the methods provided to allow replication by others?

Yes

Are the datasets clearly presented in a useable and accessible format?

Not applicable

Reviewer Expertise:

Developmental Psychology, Teratology, Pregnancy Pharmacovigilance.

I confirm that I have read this submission and believe that I have an appropriate level of expertise to confirm that it is of an acceptable scientific standard.

# Response

**Published:** 2024-07-25

**Authors:** Melissa Gladstone

**Affiliations:** University of Liverpool, UK

**Q1: The phrasing of the second sentence of the abstract "the impact of prenatal factors then influenced by postnatal environmental factors..." is not very clear.** **Maybe something along the lines of "prenatal factors and their interaction with postnatal environmental factors..." would be better.**

Response: ****We acknowledge the reviewer’s suggestion to enhance the clarity of this paragraph. We have now revised the sentence to read: *“The impact of prenatal factors and their interplay with postnatal environmental factors….”*

**Q2: In the third paragraph of the background section the sentence "lower cognitive scores, autism, and intellectual disability" reads as if autism and ID were lower in children of mothers with PIH, which I assume isn't what is intended?**

Response: We thank the reviewer for bringing this to our attention. We have addressed this concern by refining the sentence as follows:  …. *lower cognitive scores, as well as a higher incidence of autism spectrum disorders and intellectual disability*.

**Q3: In aims and objectives, the inclusion of Epilepsy as a condition to study seems to come out of the blue. While I agree it is an important disorder to include, it may be helpful to include some justification as to why it is included specifically**.

Response: We thank the reviewer for his valuable feedback. In response to this concern, we have now incorporated additional information in the fourth paragraph of the background section highlighting the association between placental complications and neurological conditions such as epilepsy as indicated below: *Although pregnancy complications are linked to adverse neurodevelopmental and neurological outcomes, which may include cerebral palsy, global developmental delay, and epilepsy, however, children survive without significant impairment.*

**Q4: Although it is clarified later on, it was not immediately clear to me how Aim 2 is distinct from Aim 1. It may be helpful to clarify further at this point.**

Response: We have addressed this concern by revising the aims and objectives sections as follows: *Specifically, we aim first to quantify the impact of pregnancy complications on the likelihood of having a moderate to severe neurodevelopmental disability, a visual impairment, and/or epilepsy) at the age of 2-3 years.* *Secondly, we aim to understand the relationship between pregnancy complications and neurodevelopmental outcomes at each time point and longitudinally across multiple developmental domains, including gross and fine motor development, language development, and social-emotional development.*

**Q5: For the observed mother-child interaction the authors state that due to concerns regarding the validity of the data when using cameras to film the assessments they instead chose to rate the interactions in the moment**. **In my experience, rating these interactions takes quite a bit of time and requires re-watching the interactions multiple times as part of the process and so I am a little concerned about the quality of these ratings done during the assessment. It may be that this specific rating scale is more simple than the scales I have experience with and my concern is unfounded. However, I think it would be helpful to further clarify how the team have ensured that these ratings are valid and reliable beyond the statement that the assessors were "well-trained".**

Response: We thank the reviewer for the insightful comment. We agree that rating mother-child interaction can be time-consuming and may require scrutiny to ensure accuracy. In response to the reviewer’s query, we incorporated measures to address these concerns. To ensure the quality and reliability of our ratings, we employed a thorough process involving double scoring for every 10 ^th^ assessment. Specifically, a senior research officer and the assigned research officer jointly rated the mother-child interaction and compared their scores. Any discrepancies were carefully discussed to ensure high inter-rater reliability. We believe that this rigorous quality check process enhances the validity and reliability of our ratings. We have now included this information under the Observation of mother-child interaction as indicated below: *…The OMCI tool is a simple rating scale that is measured at the time of assessment by a trained local culturally embedded observer. It measures maternal sensitivity, acceptance and responding, scaffolding, language stimulation, and mutual enjoyment, which are assessed during a short interaction…….* *…..To enhance the accuracy of the mother-child ratings, every 10 ^th^ dyad assessment was jointly evaluated by a senior research officer and a research officer. They compared their scores and discussed any discrepancies to ensure high inter-rater reliability.*

**Q6: I was also a bit unclear as to whether the follow-up element of the study was already ongoing or not. I recognise that this is clarified at the very end of the protocol but it may be helpful to have a comment earlier on in the document stating this very briefly.**

Response: We have now added this information under “data collection procedures” as indicated below: These assessments are on-going and are administered at each visit as summarised in Figure 2.

# Reviewer report: Reviewer response for version 1

**DOI:** 10.21956/wellcomeopenres.21809.r78607

**Published:** 2024-04-29

**Authors:** Kimford Meador

**Affiliations:** 1 Stanford University School of Medicine, Palo Alto, CA,, USA

Magai et al. describe a neurodevelopmental substudy protocol for the Precise-DYAD Network. This is an observational cohort study of female and male children (≈3,950) born to women (≈4,200) with and without placental disorders (pregnancy-induced hypertension, fetal growth restriction, and premature birth) previously recruited into Pregnancy Care Integrating Translational Science (PRECISE)-DYAD. Children will be assessed at six weeks to 6 months, 11-13 months, 23-25 months and 35-37 months in rural and semi-urban Gambia and Kenya. The aim to provide information regarding the neurodevelopment of infants and children born to women with and without placental complications at multiple time points during the first three years of life in two low-resource African communities.

 The Precise-DYAD Neurodevelopmental sub study is a well-designed prospective observational study with a very large cohort which will provide robust statistical power. Factors in pregnancy can affect child neurodevelopment, but our information in this regard remains incomplete. The very large majority of studies in this regard have been conducted in high income countries as opposed to low- and middle- income countries where differences in important factors and the population may influence fetal outcomes. The prospective collection of detail factors that might affect neurodevelopmental outcomes will provide the opportunity to examine the factors which can affect neurodevelopment. The measures for neurodevelopmental outcomes are well described and validated. Thus, the Precise-DYAD Neurodevelopmental sub study is a unique and important study.

 Several comments and suggestions to improve the protocol are respectfully offered.

 The first aim seeks to quantify the measurable impact of placental complications on physical and neurodevelopmental health trajectories, and the second aim seeks to understand the relationship between placental complications and neurodevelopmental outcomes across multiple developmental domains. Examples of placental complications are noted as pregnancy-induced hypertension (PIH), fetal growth restriction (FGR), and/or preterm birth (PTB). PIH can cause placental dysfunction while FGR and PTB are complications of placental dysfunction. Further, FGR and PTB can be caused by other factors than placental complications. Although placental complications can certainly affect the fetus and ultimate neurodevelopment, there does not appear to be a plan to directly examine the placentas. Given these issues, I would recommend measuring the impact of “PIH, FGR and PTB” rather than “placental complications” in aims 1 & 2.

 From the protocol, it is unclear how several factors are handled in data collection and analyses.

1. There is no mention of maternal medications; these should at least include known potential teratogens.
2. Are major congenital malformations, miscarriages and fetal death collected and assessed in the analyses?
3. How are socio-economic status and home environment measured?
4. How is maternal malnutrition determined? In this regard, folate is one of the important factors. I am not knowledgeable as to whether folic acid is routinely supplemented in women of childbearing age or if folic acid is routinely given once a woman is pregnant in Gambia and Kenya. It appears that folic acid intake in the first 12 weeks of pregnancy is important in child neurodevelopmental outcomes.
5. Breastfeeding can positively affect both maternal and child outcomes which affect neurodevelopment. Is this factor assessed?

Minor: Suggest rewording this sentence “Brand et al., 2020 followed a Swedish cohort of children whose mothers had PIH and found that the offspring of these mothers had significantly lower cognitive scores, autism spectrum disorders and intellectual disability.”

 To read: “Brand et al., 2020 followed a Swedish cohort of children whose mothers had PIH and found that the offspring of these mothers had significantly lower cognitive scores, and increased autism spectrum disorders and intellectual disability.”

 Minor: On page 8 in the Discussion, the abbreviation “SDG” is used which has not been previously defined.

Is the study design appropriate for the research question?

Yes

Is the rationale for, and objectives of, the study clearly described?

Yes

Are sufficient details of the methods provided to allow replication by others?

Partly

Are the datasets clearly presented in a useable and accessible format?

Not applicable

Reviewer Expertise:

Behavioral neurology, epilepsy, and neurodevelopmental effects of antiseizure medications and other factors

I confirm that I have read this submission and believe that I have an appropriate level of expertise to confirm that it is of an acceptable scientific standard, however I have significant reservations, as outlined above.

# Response

**Published:** 2024-07-25

**Authors:** Melissa Gladstone

**Affiliations:** University of Liverpool, UK

**Q1. The first aim seeks to quantify the measurable impact of placental complications on physical and neurodevelopmental health trajectories, and the second aim seeks to understand the relationship between placental complications and neurodevelopmental outcomes across multiple developmental domains. Examples of placental complications are noted as pregnancy-induced hypertension (PIH), fetal growth restriction (FGR), and/or preterm birth (PTB). PIH can cause placental dysfunction while FGR and PTB are complications of placental dysfunction. Further, FGR and PTB can be caused by other factors than placental complications. Although placental complications can certainly affect the fetus and ultimate neurodevelopment, there does not appear to be a plan to directly examine the placentas.** **Given these issues, I would recommend measuring the impact of “PIH, FGR and PTB” rather than “placental complications” in aims 1 & 2.** 

Response: We thank the reviewer for the insightful suggestions; we have now changed “placental complications” to “pregnancy complications”, defined as PIH, FGR, or PTB. We have incorporated this change throughout the paper and in the objective section as outlined below: *Specifically, we aim first to quantify the impact of pregnancy complications on the likelihood of having a moderate to severe neurodevelopmental disability, a visual impairment, and/or epilepsy at the age of 2-3 years.* *Secondly, we aim to understand the relationship between pregnancy complications and neurodevelopmental outcomes at each time point and longitudinally across multiple developmental domains, including gross and fine motor development, language development, and social-emotional development.*

**Q2: From the protocol, it is unclear how several factors are handled in data collection and analyses.**

Response: **​​​​​**We have added a dedicated section in the paper under “Assessment of Risk Factors” that details the data collection methods for all risk factors. At this stage, we will consider all the potential covariates (independent variables) that might influence the outcome. These will include variables that we know may influence child development, such as sex, socioeconomic status, maternal education, and family care indicators (stimulation in the home). For the analysis of risk factors, we have outlined the specific type of analyses we will conduct below: *For objectives 3 and 4 we will also use structural equation modelling to understand the relationships between biological, environmental, and psychosocial factors on child development at age 3. Specifically, path analysis will be used to examine the hypothesised relationship and estimate the significance and magnitude of the hypothesised causal relationship*.

**Q3: There is no mention of maternal medications; these should at least include known potential teratogens.**

Response: We thank the reviewer for these suggestions; we have now added a section describing information on maternal medication in the protocol as follows: *Mothers were asked if they were taking any medication for blood pressure, diabetes, thyroid supplements, anti-seizure medication, antiretroviral, antibiotics, or aspirin during pregnancy* *. * We have also included maternal medication in the conceptual framework (Figure 1).

**Q4: Are major congenital malformations, miscarriages and fetal death collected and assessed in the analyses?**

Response: In the initial study (PRECISE), during delivery, we collected information on stillbirth and perinatal causes of death, including congenital malformation, birth trauma, low birth weight, infection, meconium aspiration, respiratory distress, convulsions and disorders of cerebral status, prematurity, or cardiorespiratory disorders. This will all be clearly outlined in the CONSORT diagrams to make clear the number of miscarriages, stillbirths and children who were born with congenital in relation to certain pregnancy complications. However, we, of course, could not ascertain the neurodevelopment outcomes of stillbirth babies.

**Q5: How are socio-economic status and home environment measured?**

Response: The caregivers' socio-economic status was assessed using a questionnaire administered to the mothers. This information is now provided in the paper as indicated below: *The caregivers' socio-economic status was assessed using the Grameen Progress out of Poverty Index, a tool that can quantify  the share of program participants living below the poverty line, assess the performance of the intervention among the poor, and track poverty levels over time* *^1^* *. The information collected included household income, household expenditures, antenatal care expenses, antenatal admission costs, pregnancy admission costs, delivery expenses, postnatal care expenses, and health expenditures.*

**Q6: How is maternal malnutrition determined?**

Response: In this regard, folate is one of the important factors. I am not knowledgeable as to whether folic acid is routinely supplemented in women of childbearing age or if folic acid is routinely given once a woman is pregnant in Gambia and Kenya. It appears that folic acid intake in the first 12 weeks of pregnancy is important in child neurodevelopmental outcomes. We collected information on the intake of folic acid during pregnancy in the PRECISE study. We have now added this information to our paper as follows: *…*. *comprehensive data on dietary intake and nutritional status was collected on both mothers and their infants. For women, this included questionnaire-based data on food security and dietary diversity and physical measures of weight, height and mid-upper arm circumference (MUAC). Biological samples have also been collected and biobanked for future analyses of biomarkers of micronutrient status, including iron, folate, iodine, and vitamins A, D and B12. For infants, this included questionnaire-based data on infant feeding practices and dietary intake and measures of weight, length, MUAC and head circumference at 6 weeks to 6 months, 12 months, 24 months, and 36 months of age.*

**Q7: Breastfeeding can positively affect both maternal and child outcomes which affect neurodevelopment. Is this factor assessed?**

Response: Yes, we collected information on breastfeeding. We asked the mother whether the child was breastfeeding, and this information is now added to the paper as indicated below: *…* *Questionnaire-based data were collected on breastfeeding practices, including the age of cessation of exclusive breastfeeding and types of weaning foods given. Mothers were also asked whether their babies were breastfeeding*.

**Q8: Minor: Suggest rewording this sentence “Brand et al., 2020 followed a Swedish cohort of children whose mothers had PIH and found that the offspring of these mothers had significantly lower cognitive scores, autism spectrum disorders and intellectual disability.”** **To read: “Brand et al., 2020 followed a Swedish cohort of children whose mothers had PIH and found that the offspring of these mothers had significantly lower cognitive scores, and increased autism spectrum disorders and intellectual disability.”**

Response: We have revised this part as follows: *Brand et al., 2020 followed a Swedish cohort of children whose mothers had PIH and found that the offspring of these mothers had significantly lower cognitive scores, as well as a higher incidence of autism spectrum disorders and intellectual disability.*

**Q9: Minor: On page 8 in the Discussion, the abbreviation “SDG” is used which has not been previously defined.**

Response: We have spelt SDG in full as follows: *As the Sustainable Development Goals (SDG) shift focus from solely survival to surviving and thriving….*
